# Key causes and long-term trends related to emergency department and inpatient hospital admissions of homeless persons in England

**DOI:** 10.1186/s12245-023-00526-9

**Published:** 2023-08-07

**Authors:** Vibhu Paudyal, Neha Vohra, Malcolm Price, Zahraa Jalal, Karen Saunders

**Affiliations:** 1 College of Medical and Dental Sciences, University of Birmingham, Birmingham, B15 2TT UK; 2 NIHR Birmingham Biomedical Research Centre, University Hospitals Birmingham, Birmingham, UK; 3 Department of Health and Social Care, Birmingham, UK

**Keywords:** Homelessness, Inpatient admissions, Emergency department visits, Health disparity

## Abstract

**Background:**

It is estimated that approximately 300,000 people are experiencing homelessness in England. The aim of this study was to evaluate key causes and long-term trends of emergency departments (EDs) and in hospital inpatient admissions of persons experiencing homelessness in England.

**Methods:**

ED and hospital inpatient admissions data were obtained from Hospital Episode Statistics (HES) covering all National Health Service (NHS) England hospitals. Anyone identified or declared to be experiencing homelessness during the service usage are recorded in HES datasets. Data were extracted for the 10-year study period and compared to the general population, which includes all patients attending the ED or admitted to inpatient care in England.

**Results:**

Drug- and alcohol-related causes contribute to the most frequent reasons for attendance and admissions of persons experiencing homelessness in the ED and inpatient respectively. A total of 30,406 ED attendances were recorded for persons experiencing homelessness in the year 2018/2019 (+ 44.9% rise vs 2009/10) of which injuries and poisoning respectively represented 21.8% and 17.9% of all persons experiencing homelessness presentations to the ED. Poisoning (including drug overdose) represented only 1.9% of all attendances by the general population during the same study year (rate ratio vs general populations 9.2 95% *CI* 9.0–9.4). High mortality rates were observed in relation to presentations attributed to drug- and alcohol-related causes. A total of 14,858 persons experiencing homelessness inpatient admissions were recorded in 2018/2019 (+ 68.6% vs 2009/2010). Psychoactive substance use constituted 12.7% of all admissions in 2018/2019 compared to 0.4% of in the general populations (rate ratio: 33.3, 95% *CI*: 31.9–34.7). There was a 44.3% rise in the number of admissions related to poisoning in the study period amongst persons experiencing homelessness in England (vs 14.2% in general population).

**Conclusion:**

Marked disparities around primary causes of ED and inpatient admissions were identified between persons experiencing homelessness and the general population. There is a continued need for prevention measures to reduce the prevalence of drug and alcohol, injury and poisoning-related admissions to the ED, enhanced service provision at the community level, and multisector collaborations. These initiatives should maximise opportunities for early interventions and improve outcomes for persons experiencing homelessness, including increased accessibility of healthcare and mental health services, particularly in areas that demonstrate increasing ED and inpatient attendance rates over time.

## Background

Over 300,000 people are known to be currently experiencing homelessness in England [1]. Homelessness includes rooflessness (without a shelter of any kind, sleeping rough), houselessness (with a place to sleep but temporary in institutions or shelter), living in insecure housing (threatened with severe exclusion due to insecure tenancies, eviction, domestic violence), or living in inadequate housing (in caravans on illegal campsites, in unfit housing, in extreme overcrowding) [2].

While early deaths and mortality causes in persons experiencing homelessness are well reported in the literature, long-term data trends, with regard to disease epidemiology and factors that require hospitalisations and urgent care needs, have been less well documented. Systematic reviews of international literature suggest that the health status of persons experiencing homelessness is lower than the rest of the population. Persons experiencing homelessness have 12 times higher mortality rate compared with the general population, mainly owing to opioid overdose, psychoactive substance use and related heart failure [3, 4]. As per recent estimates, persons experiencing homelessness die at an average age of 46 (male) and 43 (female) years, with drug overdose and accidents contributing to the excess mortality [1]. Health status worsens with the increasing length of time spent homeless [5]. In an attempt to mitigate the negative impacts of homelessness, policy initiatives such as the Homelessness Reduction Act 2017 and the National Health Service (NHS) Long-Term Plan in England have aimed to improve outcomes for persons experiencing homelessness. These include providing support to those who are homeless or at risk of being homeless and increasing access to integrated, tailored services for rough sleepers [6, 7].

Persons experiencing homelessness present more often to emergency departments (EDs) than the general population. There is a lack of high-quality studies in England exploring the health conditions, demography, management and discharge outcomes in relation to such presentations. Many use hospitals as their only source of healthcare as they find primary care ‘complex’ to navigate, experience barriers when accessing services and have negative experiences of service use [8]. To our knowledge, long-term data trends around utilisation of ED and inpatient admissions by homeless populations have not been previously explored. Obtaining such data is imperative to identify, strengthen and evaluate appropriate primary care, community and outreach-based prevention programmes and health policies.

Due to overlapping prevalence of substance misuse and severe mental health problems associated with homelessness, much of the current healthcare focus remains on presentations related to these conditions. However, previous studies have demonstrated that important long-term health conditions, such as cardiovascular diseases amongst homeless populations, are often underdiagnosed and undertreated [9]. This study analysed ED and inpatient utilisation by persons experiencing homelessness in England and compared this with persons experiencing homelessness datasets relating to the general population. Previous research has shown that drug- and alcohol-related conditions are amongst the most frequent reasons for presentation to the ED amongst persons experiencing homelessness [10]; therefore, specific analysis explored drug- and alcohol-related presentations and admissions in detail. Using in-depth analysis of hospital episode statistics datasets from England, this study compares causes of ED and inpatient admissions by homeless populations between 2009/2010 and 2018/2019.

## Material and methods

### Study design

This study used retrospectively collected routine data from government sources pertaining to the EDs and inpatient service utilisation and outcomes relating to persons experiencing homelessness in England. General population data relating to all other patients attending the ED or admitted to inpatient care in England, available from NHS Digital [11, 12], was used as the comparator.

### Setting

Data were extracted from the Hospital Episode Statistics, a routinely collected data source relating to EDs and inpatient admissions. Data specific to persons experiencing homelessness was requested through a data procurement agreement between University of Birmingham and NHS Digital. Persons experiencing homelessness-specific data were identified by searching patient records for specific postcode fields that are used to record homelessness when patients with no fixed abode present to EDs and inpatient services. Datasets from 10 years (2009/2010 to 2018/2019) were extracted and analysed. ED attendance and inpatient admissions data relevant to all English general populations over the study period were used as a comparator population.

### Data extraction

#### ED admissions data

Counts of all attendance over the study period and specific to the two-character level primary diagnosis codes were extracted for both persons experiencing homelessness and the general populations. In addition, data on age category, sex, ethnicity (White, non-White and unknown), referral methods (e.g. emergency services, general practitioner (GP), local authority, police), arrival modes (ambulance, other, unknown), admissions methods (elective, emergency and other) and attendance disposal methods (to hospital; died, other and unknown) were extracted.

#### Inpatient admissions data

A count of all consultant episodes over the study period and specific to the three-character level primary diagnosis codes as per the International Classification of Diseases (ICD) codes were extracted for both persons experiencing homelessness and the general populations. In addition, data on age category, ethnicity (White, non-White and unknown), admissions methods (elective, emergency, other), discharge methods (e.g. through clinical consent, self-discharged, still in hospital) and disposal methods (i.e. dead, alive and not known) were extracted.

### Data collection and management

Data search was run by NHS Digital, the national information and technology partner to the health and social care system. All data were anonymised, and small numbers suppressed to protect the anonymity and confidentiality prior to release to the research team. All evaluation materials were stored, processed and destroyed in accordance with University of Birmingham research governance policies. This study was reviewed and approved by Data Access Request Service (DARS) Review Committee of NHS Digital (approval reference number NIC-341255-H2F7H).

### Analysis

Histograms presenting the numbers of persons with each diagnosis were plotted for all included persons. For all included persons, the number and percentage of all key causes of ED presentations and inpatient admissions were descriptively presented. In depth analyses of poisoning, drug- and alcohol-related causes were separately presented. All persons experiencing homelessness data from England were compared to the general population ED attendance and inpatient admission datasets. A time trend plot was constructed to illustrate the changes in ED presentations and inpatient admissions pattern over the 10-year study period. Comparisons between years were performed using ratios of proportions and 95% confidence intervals.

## Results

### Emergency department presentations

There were a total of 44,061 ED presentations made by persons experiencing homelessness in England in 2018/2019, representing an increase of 44.9% from 2009/2010 in which 30,406 visits were recorded. An increase of 77.4% was observed in the general population in England, from approximately 9 million visits in 2009/2010 to 16 million visits in 2018/19 (Table 1).

**Table 1:** All causes of ED presentations by persons experiencing homelessness (PEH)

**Diagnosis description**	**England (number of admissions) **					
	**Homeless persons**			**General population**		
	**2009/2010**	**2018/2019**	**% difference**	**2009/2010**	**2018/2019**	**% difference**
All injuries	9561	9291	− 2.8	3,912,758	5,859,043	49.7
Poisoning (inc. overdose)	2996	7876	162.9	127,240	311,370	144.7
Infectious disease	190	520	173.7	102,574	265,173	158.5
Local infection	774	1440	86.0	220,534	349,308	58.4
Septicaemia	18	141	683.3	9080	112,169	1135.3
Cardiac conditions	764	779	2.0	334,200	645,822	93.2
Cerebrovascular conditions	175	167	− 4.6	94,412	233,811	147.6
Other vascular conditions	131	329	151.1	46,877	81,845	74.6
Haematological conditions	79	124	57.0	26,327	98,472	274.0
Central nervous system conditions (exc stroke)	1062	768	− 27.7	214,624	243,252	13.3
Respiratory conditions	854	1738	103.5	411,149	1,119,302	172.2
Gastrointestinal conditions	1243	1635	31.5	511,533	1,019,153	99.2
Urological conditions (inc. cystitis)	435	719	65.3	197,414	628,136	218.2
Diabetes and other endocrinological conditions	121	243	100.8	44,356	89,790	102.4
Allergy (inc. anaphylaxis)	119	124	4.2	55,838	98,712	76.8
Psychiatric conditions	1608	3917	143.6	90,079	267,592	197.1
Social problems (inc. chronic alcoholism and homelessness)	869	1072	23.4	29,620	53,578	80.9
Diagnosis not classifiable	5900	8098	37.3	1,565,348	1,988,773	27.0
Nothing abnormal detected	1583	3287	107.6	312,994	1,061,415	239.1
**Total**	**30,406**	**44,061**	**44.9**	**9,043,559**	**16,040,964**	**77.4**

All numbers relate to finished consultant episodes

Injuries and poisoning respectively contributed to 21.8% (*n* = 9291) and 17.9% (*n* = 7876) of all persons experiencing homelessness presentations to the ED in 2018/2019. While injury was the most common cause of presentation to the ED by the general population (36.5%) (*n* = 5,859,043), respiratory, gastrointestinal and cardiac conditions were the other most common causes (Table 1). Poisoning (including drug overdose) represented only 1.9% (*n* = 311,370) of all attendances by the general population during 2018/2019 (rate ratio compared to persons experiencing homelessness vs general populations 9.21, 95% *CI* 9.02–9.40).

**Fig. 1:**
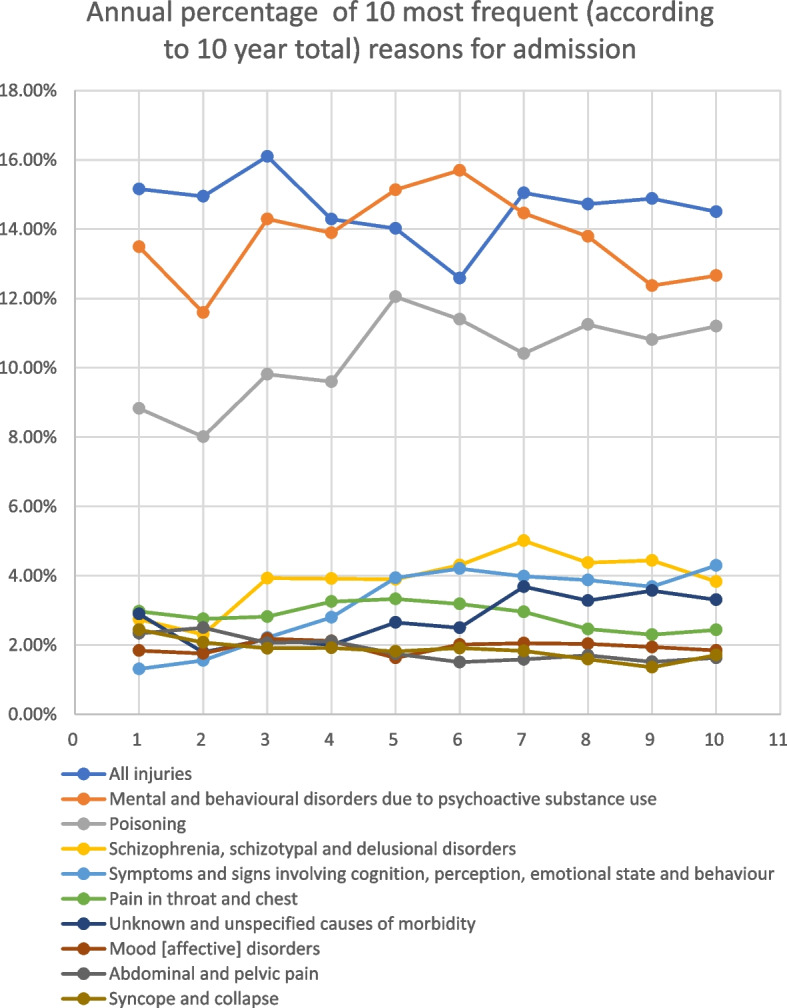
Time-trend analysis of key causes of emergency department presentations for the period covering 2009/2010 to 2018/2019. Datapoints 1, 2000/2010; 2, 2010/2011; 3, 2011/2012; 4, 2012/2013; 5, 2013/2014; 6, 2014/2015; 7, 2015/2016; 8, 2016/2017; 9, 2017/2018; 10, 2018/2019

Persons experiencing homelessness attendance for poisoning (including overdose) increased by 163% (*n* = 2996 in 2009/2010 vs *n* = 7876 in 2018/2019), and attendance by the general population increased by 145% (*n* = 127,240; *n* = 311,370) during the study period (Table 2, Fig. 1). The total consultation for drug and alcohol problems in people experiencing homelessness saw an 44.9% increase from 3865 in 2009/2010 to 8948 attendance in 2018/2019. Whereas in the general populations, the increase was 132.7% with numbers increasing from 156,860 in 2009/2010 to 364,948 in 2018/2019. Other conditions that saw an increase in admissions amongst persons experiencing homelessness related to infectious disease (174%), septicaemia (683%), other vascular conditions (151%), respiratory conditions (104%), diabetes and other endocrinological conditions (101%) and psychiatric conditions (144%). In the general population, septicaemia saw the highest increase in ED admissions of 1135% during the study period.

**Table 2:** Drug- and alcohol-related ED presentations by persons experiencing homelessness (PEH) (England)

	Diagnosis description	Finished consultant episodes (total patients)	% male		Mean age		% White ethnicity		% arrival by ambulance		% who died	
			PEH	Gen Popl	PEH	Gen Popl	PEH	Gen Popl	PEH	Gen Popl	PEH	Gen Popl
**2018/2019** Total	Poisoning (inc. overdose)	7876	83.5	NA	43.0	NA	70.4	NA	76.7	NA	20.9	NA
	Social problems (inc. chronic alcoholism and homelessness)	1072	78.5	NA	44.4	NA	69.7	NA	50.4	NA	11.8	NA
	Total drug and alcohol	8948	82.9	NA	43.2	NA	70.3	NA	73.5	NA	19.9	NA
	**All disease diagnoses**	44,061	76.7	48.7	41.1	NA	68.0	70.4	43.2	21.8	0.12	0.10
**2009/2010** Total	Poisoning (inc. overdose)	2996	82.2	NA	63.0	NA	NA	NA	81.5	NA	32.7	NA
	Social problems (inc. chronic alcoholism and homelessness)	869	90.2	NA	55.0	NA	NA	NA	76.1	NA	25.0	NA
	Total drug and alcohol	3865	84.0	NA	61.2	NA	NA	NA	80.3	NA	31.0	NA
	**All disease diagnoses**	30,406	73.3	51.2	52.5	NA	NA	NA	47.9	25.2	0.37	0.20

Amongst the persons experiencing homelessness presenting with drug- and alcohol-related causes in 2018/2019, approximately 83% were male (77% males for all causes), 70.4% were of white ethnicity (68.0% for all causes), and nearly twice as many (73.5%) arrived by ambulance compared to all causes (43.2%).

Approximately, 0.12% of all persons experiencing homelessness who presented to ED during 2018/2019 died in the ED compared to 0.10% of deaths in general population (rate ratio 1.23, 95% *CI*: 0.94 to 1.60). Nearly 1 in 5 who presented with drug- and alcohol-related problems in the ED died compared to 0.1% of recorded deaths for all diagnoses in 2018/19. However, there was a reduction in mortality in the ED due to drug- and alcohol-related problems during the study period (31.0% in 2009/2010 vs 19.9% in 2018/2019).

Nearly half of all arrivals of persons experiencing homelessness in the ED were via ambulance (44.7%) compared to 22.7% in the general population (rate ratio 1.97, 95% *CI*: 1.95–1.99). A total of 5.7% (*n* = 2518) of all persons experiencing homelessness ED attendance were related to referrals by the police. This was the second most common mode of referral to the ED after self-referral (*n* = 27,652, 62.7%) in England. Seasonal variations in presentations were low. Presentations during admissions quarter 1 (April-June), quarter 2 (July–September), quarter 3 (October-December) and quarter 4 (January—March) were 25.4%, 27.0%, 24.3%, and 23.3% respectively in 2018/19.

### Adult inpatient admissions

There were a total of 14,858 (2018/19) inpatient admissions of persons experiencing homelessness recoded in England in 2018/2019, an increase of 13.8% from 2009/2010 (*n* = 13,061). In the general population in England, ED presentations increased by 23.5% from approximately 16.8 million in 2009/2010 to 20.7 million admissions in 2018/2019.

Injuries were recorded as the most prevalent cause of persons experiencing homelessness inpatient admissions in 2018/2019 (Table 3), which saw a total of 2155 admissions. This was followed by mental and behavioural disorders due to psychoactive substance use (*n* = 1881) and poisoning (*n* = 1664). Psychoactive substance use constituted 12.7% of all admissions of persons experiencing homelessness to inpatient units compared to 0.4% of admissions in the general populations (rate ratio: 33.27, 95% *CI*: 31.87–34.72]. Similarly, 11.2% of all admissions to inpatient units in 2018/2019 were contributed by poisoning compared to 0.8% contribution in the general population (rate ratio: 14.55, 95% *CI*: 13.90–15.23).

**Table 3:** Causes of inpatient admissions persons experiencing homelessness (PEH)

**Inpatient diagnosis description**	**England**					
	**Homeless persons**			**General population**		
	**2009/2010**	**2018/2019**	**% difference (2018/2019–2009/2010)**	**2009/2010**	**2018/2019**	**% difference (2018/2019–2009/2010)**
All injuries	1980	2155	8.8	750,615	848,535	13.0
Poisoning	1153	1664	44.3	139,922	159,802	14.2
Intestinal infectious diseases	30	65	116.7	68,572	212,450	209.8
Tuberculosis	6	15	150.0	7172	4771	− 33.5
Sepsis (streptococcal sepsis and other sepsis)	18	162	800.0	37,491	310,619	728.5
Viral hepatitis	3	4	33.3	6784	4006	− 40.9
Neoplasms	147	127	− 13.6	1,701,525	2,272,795	33.6
Anaemias	60	45	− 25.0	212,489	339,524	59.8
Diabetes mellitus	64	155	142.2	83,924	93,774	11.7
Mental and behavioural disorders due to psychoactive substance use	1762	1881	6.8	69,463	79,004	13.7
Schizophrenia, schizotypal and delusional disorders	358	569	58.9	35,677	38,023	6.6
Mood (affective) disorders	240	274	14.2	40,095	32,042	− 20.1
Neurotic, stress-related and somatoform disorders	100	125	25.0	18,616	26,391	41.8
Disorders of adult personality and behaviour	128	231	80.5	9732	15,301	57.2
Epilepsy	223	164	− 26.5	54,428	57,630	5.9
Hypertensive diseases	17	18	5.9	50,420	31,662	− 37.2
Ischaemic heart diseases	98	76	− 22.4	407,675	400,400	− 1.8
Pulmonary embolism	11	28	154.5	37,333	55,626	49.0
Cardiac arrest	8	13	62.5	5425	6,692	23.4
Atrial fibrillation and flutter	41	35	− 14.6	126,235	160,875	27.4
Heart failure	21	28	33.3	112,976	188,683	67.0
Cerebrovascular diseases	104	51	− 51.0	203,705	214,886	5.5
Diseases of veins, lymphatic vessels and lymph nodes, not elsewhere classified	95	191	101.1	172,999	109,196	− 36.9
Influenza and pneumonia	108	294	172.2	271,822	625,585	130.1
Other chronic obstructive pulmonary disease	79	154	94.9	181,491	246,646	35.9
Asthma	45	75	66.7	79,849	111,081	39.1
Diseases of oral cavity, salivary glands and jaws	66	61	− 7.6	269,861	266,368	− 1.3
Diseases of oesophagus, stomach and duodenum	106	126	18.9	389,741	491,793	26.2
Alcoholic liver disease	68	67	− 1.5	29,892	48,628	62.7
Other liver diseases	17	17	0.0	22,227	42,581	91.6
Cutaneous abscess, furuncle and carbuncle	58	285	391.4	34,562	53,633	55.2
Cellulitis	180	337	87.2	87,749	154,321	75.9
Ulcer of lower limb, not elsewhere classified	48	140	191.7	18,533	25,186	35.9
Pain in throat and chest	388	362	− 6.7	335,846	324,014	− 3.5
Abdominal and pelvic pain	305	242	− 20.7	328,581	389,966	18.7
Symptoms and signs involving cognition, perception, emotional state and behaviour	171	638	273.1	85,952	112,743	31.2
Syncope and collapse	319	254	− 20.4	124,516	109,803	− 11.8
Convulsions, not elsewhere classified	213	184	− 13.6	51,790	59,506	14.9
Unknown and unspecified causes of morbidity	378	491	29.9	99,721	231,102	131.7
**All disease diagnoses**	**13,061**	**14,858**	**13.8**	**16,806,196**	**20,760,699**	**23.5**

Mental health conditions such as schizophrenia, schizotypal and delusional disorders, mood (affective) disorders and cellulitis are also featured as important causes of admissions. For example, in 2018/2019, a total of 569 and 274 inpatient admissions were noted for schizophrenia, schizotypal and delusional disorders and mood (affective) disorders respectively amongst people experiencing homelessness. Another 1881 admissions were related to mental and behavioural disorders due to psychoactive substance use. In the English general population, cancers, injuries, influenza and pneumonia (3.0%), diseases of oesophagus, stomach and duodenum and anaemias and ischaemic heart diseases were featured amongst the most important causes of admissions in 2018/2019 (Table 3). Amongst these, sepsis (800%) saw the biggest increase. In the general population in England, sepsis (728.5%), influenza and pneumonia (130.1%) saw a significant increase in the number of admissions during the study period.

A total of 24.3% (3612) of all persons experiencing homelessness inpatient admissions in 2018/2019 were attributed to drug- and alcohol-related causes in England (Tables 4 and 5). Of these, a total of 1531 admissions were related to ‘mental and behavioural disorder due to the use of alcohol’ (Table 5). There was a 6.8% increase (compared to 13.7% increase in the general population in England) in the number of admissions categorised as ‘mental and behavioural disorders due to psychoactive substance use’ during the 10-year study period. This included admissions related to the use of alcohol, opioids, cannabinoids, cocaine and polysubstance use. Mental and behavioural disorders due to use of cocaine (+ 480%) and ‘other stimulants, incl. caffeine’ (360.0%), saw the highest increase in admission number amongst persons experiencing homelessness during the study period. In comparison, admissions related to mental and behavioural disorders due to use of tobacco, cocaine and cannabinoids saw the largest increase amongst the general populations in England (Table 5).

**Table 4:** Drug- and alcohol-related inpatient admissions amongst persons experiencing homelessness (PEH) in England

	**Diagnosis description**	**Finished consultant episodes (total patients)**		**% male**		**Mean age**		**% White ethnicity**		**% emergency admissions**		**% who died in inpatient**	
		**PEH**	**Gen pop**	**PEH**	**Gen popl**	**PEH**	**Gen popl**	**PEH**	**Gen popl**	**PEH**	**Gen popl**	**PEH**	**General pop**
**2018–2019**	Mental and behavioural disorders due to psychoactive substance use	1881	79,004	87.6	70.4	43.9	46.1	72.8	NA	93.1	95.0	0	NA
	Alcoholic liver disease	67	48,628	85.1	65.2	**47.5**	54.4	72.8	NA	93.1	64.3	1.5	NA
	Poisoning	1664	159,802	75.8	40.3	**36.3**	36.6	80.5	NA	99.8	97.9	0.1	NA
	Total drug and alcohol	3612	287,434	82.1	52.8	40.4	42.2	76.5	NA	96.2	93.3	0.1	NA
	**All disease diagnoses**	**14,858**	**20,760,699**	**76.9**	**45.4**	**41.6**	**53.8**	**71.4**	**NA**	**86.7**	**37.6**	**0.6**	**NA**
**2009–2010**	Mental and behavioural disorders due to psychoactive substance use	1762	69,463	86.9	70.8	43.2	42.9	76.4	NA	95.26	85.6	0	NA
	Alcoholic liver disease	68	29,892	85.3	67.2	42.4	52.0	86.8	NA	98.56	71.7	1.5	NA
	Poisoning	1153	139,922	79.4	41.8	34.5	35.6	84.4	NA	99.6	98.7	0.3	NA
	Total drug and alcohol related	2983	239,277	83.9	53.4	39.8	39.8	79.8	NA	97.0	92.8	0.1	NA
	**All disease diagnoses**	**13,061**	**16,806,196**	**75.9**	**44.1**	**39.9**	**51.0**	**73.5**	**NA**	**82.4**	**35.6**	**0.6**	**NA**

**Table 5:** Drug- and alcohol-related inpatient admissions amongst persons experiencing homelessness (PEH) in England- detailed causes

**Diagnosis code**	**Diagnosis description**	**PEH**			**General populations**		
		**2009/2010**	**2018/2019**	**% difference**	**2009/2010**	**2018/2019**	**% difference**
F10	Mental and behavioural disorders due to use of alcohol	1613	1531	− 5.1	62,511	67,903	8.6
F11	Mental and behavioural disorders due to use of opioids	46	76	65.2	2561	1510	− 41.0
F12	F12: Mental and behavioural disorders due to use of cannabinoids	21	35	66.7	835	1580	89.2
F13	F13: Mental and behavioural disorders due to use of sedatives or hypnotics	3	6	100.0	228	336	47.4
F14	F14: Mental and behavioural disorders due to use of cocaine	5	29	480.0	425	916	115.5
F15	F15: Mental & behav’l disord’s due to use of other stimulants, incl. caffeine	5	23	360.0	406	738	81.8
F16	F16: Mental and behavioural disorders due to use of hallucinogens	2	1	− 50.0	120	151	25.8
F17	F17: Mental and behavioural disorders due to use of tobacco	1	1	0.0	68	786	1055.9
F18	F18: Mental and behavioural disorders due to use of volatile solvents		1		39	17	− 56.4
F19	F19: Mental & behav’l disorders due to multiple drug/psychoactive subuse	66	178	− 169.7	2270	5067	123.2
**Total F10–F19**	**Mental and behavioural disorders due to psychoactive substance use**	**1762**	**1881**	**6.8**	**69,463**	**79,004**	**13.7**
**K70**	**Alcoholic liver disease**	**68**	**67**	− **1.5**	**29,892**	**48,628**	**62.7**
T36	Poisoning by systemic antibiotics	10	6	− 40.0	1020	626	− 38.6
T37	Poisoning by other systemic anti-infectives and antiparasitics	2	3	50.0	471	320	− 32.1
T38	Poisoning by hormone and synthetic substitute and antagonists, NEC	11	66	500.0	2749	4114	49.7
T39	Poisoning by nonopioid analgesics, antipyretics and antirheumatics	382	425	11.3	57,687	61,934	7.4
T40	Poisoning by narcotics and psychodysleptics (hallucinogens)	230	484	110.4	12,496	22,755	82.1
T41	Poisoning by anaesthetics and therapeutic gases	8	5	− 37.5	329	357	8.5
T42	Poisoning by antiepileptic, sedative-hypnotic & antiparkinsonism drugs	187	234	25.1	18,605	17,275	− 7.1
T43	Poisoning by psychotropic drugs, not elsewhere classified	200	265	32.5	23,367	28,554	22.2
T44	Poisoning by drug, primarily affecting the autonomic nervous system	4	25	525.0	2267	3498	54.3
T45	Poisoning by primarily systemic and haematological agents, NEC	14	15	7.1	2742	3278	19.5
T46	Poisoning by agents primarily affecting the cardiovascular system	6	5	16.7	2199	2388	8.6
T47	Poisoning by agents primarily affecting the gastrointestinal system	4	1	− 75.0	576	598	3.8
T48	Poison’g by agents prim act’g on smooth & skeletal muscles & resp system	1	1	0.0	433	388	− 10.4
T49	Pois top agt prim affect skin muc memb & by ophth’l, oto’l & dental drugs	2	1	− 50.0	593	463	− 21.9
T50	Poison’g by diuretics & other unspec’d drugs medic’ts & biol’l subs	54	85	57.4	5379	3819	− 29.0
T51	Toxic effect of alcohol	15	28	86.7	1546	1450	− 6.2
T52	Toxic effect of organic solvents	2	3	− 50.0	610	748	22.6
T53	Toxic effect of halogen derivatives of aliphatic & aromatic hydrocarbons	-	-	-	17	28	64.7
T54	Toxic effect of corrosive substances	5	2	− 60.0	801	1204	50.3
T55	Toxic effect of soaps and detergents				192	230	19.8
T56	Toxic effect of metals	1	1	0.0	435	536	23.2
T57	Toxic effect of other inorganic substances				13	15	15.4
T58	Toxic effect of carbon monoxide	3	3	0.0	551	296	− 46.3
T59	Toxic effect of other gases, fumes and vapours	7	5	− 28.6	1643	1114	− 32.2
T60	Toxic effect of pesticides	2		-	212	207	− 2.4
T61	Toxic effect of noxious substances eaten as seafood	-	-	-	62	33	− 46.8
T62	Toxic effect of other noxious substances eaten as food	-	-	-	444	211	− 52.5
T63	Toxic effect of contact with venomous animals	-	-	-	1783	2698	51.3
T65	Toxic effect of other and unspecified substances	3	1	− 66.7	700	665	− 5.0
**Total: T36–T65**	**Poisoning**	**1153**	**1664**	**44.3**	**139,922**	**159,802**	**14.2**
	Total drug and alcohol	2983	3612	21.1	239,277	287,434	20.1
	**All disease diagnoses**	**13,061**	**14,858**	**13.8**	**16,806,196**	**20,760,699**	**23.5**

There was a 44.3% rise in the number of admissions related to poisoning amongst persons experiencing homelessness in England during the study period (compared to 14.2% rise in the general population) (Tables 4 and 5). Poisoning included toxic effects of alcohol, drugs of abuse, prescribed drugs with no abuse potential and corrosive substances and other known poisons.

### Self-discharge without clinical consent

A total of 3415 out of 14,858 (23.0%) persons are experiencing homelessness self-discharged (i.e. without clinical consent) from the inpatient units in 2018/2019. The data was not available to compare this figure with the general population.

## Discussion

### Summary of key findings

The findings of this study provide an overview of the secondary healthcare utilisation by persons experiencing homelessness in England over the 10-year study period. Comparisons were drawn with data from the general population covering all ED attendance and inpatient admissions.

Cases of poisoning contributed to over 1 in 5 presentations to the ED by persons experiencing homelessness. The observed rate was over 10 times higher compared to the rates in the general population. Of note, during the 10-year study period, poisoning-related attendances amongst persons experiencing homelessness increased by over 160% in England. The rate of increase was higher than those observed in the English general population.

This study shows that drug- and alcohol-related admissions contributed to high mortality in the ED (approximately 20%) compared to 0.1% for all diagnoses. However, over the study period, mortality rates in the ED due to drug- and alcohol-related causes saw significant reductions.

Psychoactive substance use contributed to the most frequent cause of inpatient admissions in persons experiencing homelessness. Key causes of admissions were markedly different when compared with the general population. In the latter, cancers, respiratory and cardiovascular causes dominated the key causes of admissions. Causes such as sepsis, leg ulcers and diabetes saw the largest increase in the number of persons experiencing homelessness inpatient admissions during the study period. Furthermore, for mental health, drug- and alcohol-related diagnoses such as schizophrenia, personality disorders and poisoning, the rate of increase was markedly higher in persons experiencing populations. These findings corroborate with persons experiencing homelessness mortality data as reported by the Office of the National Statistics in England which demonstrate that drug- and alcohol-related conditions are amongst the most significant causes of mortality [5].

### Study strengths and limitations

This is the first study evaluating the causes of ED presentations and inpatient admissions by persons experiencing homelessness using nationally collected datasets from England. Consideration of 10 years of data allowed time trends to be analysed. It is important to note that recording of homelessness is often not complete or accurate in emergency departments and hospital inpatient settings. We relied on the postcode fields used to record the domicile of anyone with no fixed abode when they present for services. Therefore, the data presented in this report may not represent all attendance and outcomes of persons experiencing homelessness in the two study settings. Changes in homelessness numbers, recording practices and evolution of services, particularly those aimed at persons experiencing homelessness, need to be taken into consideration when interpreting the time trend data. For example, lately, there is an increasing emphasis on the identification of persons experiencing homelessness in the hospital and primary care settings since the introduction of the Homelessness Reduction Act [6]. In addition, there is a lack of accurate data in regards to the number of persons experiencing homelessness in England as official sources often refer to the ‘households’ [13]. We were unable to estimate the incident rates of persons experiencing homelessness utilising ED and inpatient due to the aforementioned factors. It is important to note that homelessness has increased in England. Given the lack of accurate figures on the number of experiencing homelessness as described above and by instead using measures of households (not individuals) in temporary accommodation, there has been a 65.7% increase in homelessness in England during the study period 2010 (*n* = 51,310) v 2019 (*n *= 85,040) [13]. Therefore, the time trend estimates in relation to persons experiencing homelessness presentations should be interpreted accordingly. The link between homelessness and substance and/or alcohol dependence is well understood in the literature; however, this study allowed all key health conditions to be systematically examined in relation to their contribution.

Although the presentations and admissions due to nondrug- and alcohol-related causes were lower amongst persons experiencing homelessness, literature demonstrates that they experience higher burden of multimorbidity and mortality rates attributed to these conditions compared to the general population [5, 14].

### Implications for practice

This study reinforces the need to improve the provision of mental health and substance misuse-related support to persons experiencing homelessness in the community. As is well rehearsed, prevention measures should be further strengthened to address the health inequalities faced by this population.

Previous studies show that persons experiencing homelessness are underrepresented in the mainstream practices [15]. While specialist primary healthcare centres for homeless persons have been established, mainstream services need to be further inclusive of persons experiencing homelessness. In addition, outreach-based and community services are best able to serve this population [16–20]. Training and education of frontline staff at mainstream general practices are required to reinforce the registration guidelines [21]. Entry criteria to primary care, mental health and substance misuse services for persons experiencing homelessness need to be reviewed in order to increase accessibility. As indicated in the Public Health England Guidance [22] providers of alcohol and drug, mental health and other services need to adopt an open-door policy for individuals with co-occurring conditions and should ensure every contact opportunity counts. Treatment for any of the co-occurring conditions should be available through every contact point. Services such as needle exchange and naloxone provision need to be readily available in the community, including through community pharmacies. It has been reported that the COVID-19 pandemic impacted heavily on provision of drug and alcohol services as well as those serving the homeless populations [23, 24]. It is important that lessons and good practices from the pandemic are considered for future service provision. These include the use of remote- and technology-assisted services to serve the affected populations which were reported to have been well received by the clients [23, 24].

There is also a requirement for compliance with the Homelessness Reduction Act 2017 [6] to ensure healthcare settings, particularly hospitals, proactively identify persons experiencing homelessness and work collaboratively with social services to offer support to them or those threatened with homelessness. Furthermore, this study has reinforced the need to adhere to the goals set out in the NHS Long-Term Plan in order to provide outreach services and invest in mental health support for persons experiencing homelessness. A comprehensive health needs assessment tool, for use by primary care, mental health, substance misuse and public health practitioners, is needed in order to support practitioners and increase their confidence when addressing complex issues.

Future studies should consider accessing individual medical notes and health-related data from multiple sources to triangulate the findings. In this study, it was not possible to investigate repeat ED attendance by a person due to lack of identifiable personal data and postcodes. There is a need for research investigating repeat attendance and associated reasons and in addition to develop integrated models of care to address multiple morbidities including overlapping substance misuse and mental health [25–27]. Published literature report that persons experiencing homelessness are likely to attend the ED far more than the general population, with wide variations in the figures reported [28]. For example, persons experiencing homelessness have been reported to use the ED 5 [29], 20 [30] or 60 [14] times more compared to the general population.

## Conclusion

Poisoning and drug- and alcohol-related causes contribute to the most frequent reasons for attendance and admissions of persons experiencing homelessness to the ED and inpatient settings. The key causes of healthcare utilisation differ markedly from the general population data. There is a need to increase outreach-based services and support and review entry criteria to primary care mental health and substance misuse services for persons experiencing homelessness. There is a continued need for prevention measures, development of outreach-based support and enhanced service provision at the community level. Multisector collaborations are needed to maximise opportunities for early interventions. The data presented here relates to pre-COVID-19 pandemic timelines, and further research is required to assess the wider impacts of the pandemic on homelessness in England. A longitudinal evaluation will enable the identification of how the above data trends have changed as a result of the pandemic and support provisions made by the government and local authorities to alleviate the impact of the pandemic on persons experiencing homelessness.

## Data Availability

The datasets used and/or analysed during the current study are available from the corresponding author on reasonable request.

## Abbreviations

DARS: Data Access Request Service
GP: General practitioner
HES: Hospital Episode Statistics
ICD: International Classification of Diseases
NHS: National Health Service
PEH: Persons experiencing homelessness
